# Biofilm infection of a central venous port-catheter caused by *Mycobacterium avium* complex in an immunocompetent child with cystic fibrosis

**DOI:** 10.1186/s12879-022-07899-x

**Published:** 2022-12-09

**Authors:** Alexandra Kavvalou, Florian Stehling, Eva Tschiedel, Jan Kehrmann, Bernd Walkenfort, Mike Hasenberg, Margarete Olivier, Mathis Steindor

**Affiliations:** 1 Department of Pediatric Pulmonology and Sleep Medicine, University Hospital Essen, Children’s Hospital, University of Duisburg-Essen, Hufelandstr. 55, 45147 Essen, Germany; 2 Department of Pediatric Intensive Care, Children’s Hospital, University of Duisburg-Essen, Essen, Germany; 3 Institute of Medical Microbiology, University Hospital Essen, University of Duisburg-Essen, Essen, Germany; 4 Institute for Experimental Immunology and Imaging, Imaging Center Essen, Electron Microscopy Unit (EMU), University of Duisburg-Essen, Essen, Germany

**Keywords:** MAC, Biofilm infection, Cystic fibrosis

## Abstract

**Background:**

*Mycobacterium (M.) chimaera* is a non-tuberculous mycobacterium (NTM) that belongs to *M. avium complex* (MAC). In patients with cystic fibrosis (CF), MAC can cause bronchopulmonary infections that can be prolonged and difficult to treat. MAC infections of sites other than the lungs or central catheters are rare and almost exclusively associated with immunodeficiency.

**Case presentation:**

We present a case of an 8-year-old CF patient (delF508 homozygous) with recurrent pulmonary exacerbations, gradual clinical deterioration, B-symptoms (fever, fatigue, weight loss, night sweat), elevated transaminases and intermittent detection of *M. chimaera* in the sputum without radiological signs of NTM-associated lung disease with a central venous port-catheter. Next-generation sequencing (NGS) revealed *M. chimaera* port infection that was also confirmed by mycobacterial culture. The patient recovered within 4 weeks after removal of the catheter and initiation of MAC targeted antimicrobial therapy. Electron microscopy of the catheter illustrated the presence of mycobacteria in a biofilm.

**Conclusions:**

MAC central venous catheter infection needs to be considered in immunocompetent people. NGS is a valuable tool for rapid identification of rare infections. MAC capability of biofilm formation renders catheter removal the central therapeutic intervention for the clearance of the infection.

## Background

*Mycobacterium chimaera* is a species of slow-growing non-tuberculous mycobacteria (NTM) and part to the *Mycobacterium avium* complex (MAC) [1]*. M. chimaera* is a biofilm-producer [2], recovered from the environment including household water and may act as an opportunistic pathogen in immunocompromised patients [3]. It has been identified as causative pathogen for several diseases, including pneumonia, endocarditis, chorioretinitis, hepatitis, pancytopenia as well as disseminated infection [4]. This species has particularly gained attention as causative agent for endocarditis and disseminated disease in post-operative cardiac patients after exposure to heater-cooler units in several countries [5, 6]. Histologically, infections are characterized by granulomatous lesions, which can mislead to other diagnoses (e.g. sarcoidosis) [7].

However, the pathogenicity of MAC is limited, as MAC predominantly causes localized infections, especially of the lung. In Cystic Fibrosis (CF), *M. chimaera* and other MAC species are increasingly recognized as cause of lung infections, reaching a prevalence up to 9% [8, 9]. Globally, MAC is the most frequently isolated NTM from CF respiratory specimens [10]. Notably, MAC detection in CF sputum does not per se define NTM lung disease and may be transient and harmless [11, 12]. In fact, invasive lung disease appears to affect less than 50% of MAC positive people with CF (pwCF) [5, 13]. In pwCF, MAC lung infections have been thoroughly documented and investigated, but MAC infections of other sites or catheters are not reported.

## Case presentation

An 8-year-old Caucasian female patient with CF (delF508 homozygous), with an implanted port-catheter and under CFTR-modulator therapy (lumacaftor/ivacaftor) presented with recurrent pulmonary exacerbations, B-symptoms including fever, night sweat, fatigue and weight loss. Laboratory work-up revealed elevated aminotransferases, which persisted despite discontinuation of CFTR modulator therapy (max. AST 370 U/l, ALAT 440 U/l). In parallel, the patient presented a decline of her baseline lung function (undulating around ppFeV1 60%, ppFeV1 min. 35%, baseline 81%). *Mycobacterium chimaera* was intermittently recovered from sputum cultures (5 out of 17 sputum cultures in 7 months), while chest computed tomography showed no NTM-lung disease defining pattern. The conventional sputum cultures in parallel revealed repeatedly a polymicrobial infection with multiple CF-typical pathogens (*Achromobacter sp., Stenotrophomonas maltophilia, Pseudomonas putida, Haemophilu influenzae, Aspergillus fumigatus, Staphylococcus aureus),* so bronchoscopy with bronchoalveolar lavage was not performed. Over this period of 7 months, the patient was hospitalized 4 times for intravenous antibiotic therapy (meropenem, piperacillin/ tazobactam and vancomycin) and received 4 courses of oral antibiotics (amoxicillin/ clavulanic acid, co-trimoxazole, doxycycline, ciprofloxacin), each course for 10–21 days. No antibiotic with marked activity against MAC was administered. All antibiotic therapies resulted in partial temporary improvement of the clinical condition and the lung function. All conventional blood culture bottles drawn for the detection of aerobic and anaerobic bacteria remained sterile, the inflammation parameters were mildly elevated or negative (CrP max. 5.8 mg/dl, procalcitonin max. 2.74 ng/ml). The liver enzymes remained elevated and the subsequent liver biopsy revealed granulomatous hepatitis that was attributed to lumacaftor/ivacaftor toxicity at that time.

**Fig. 1:**
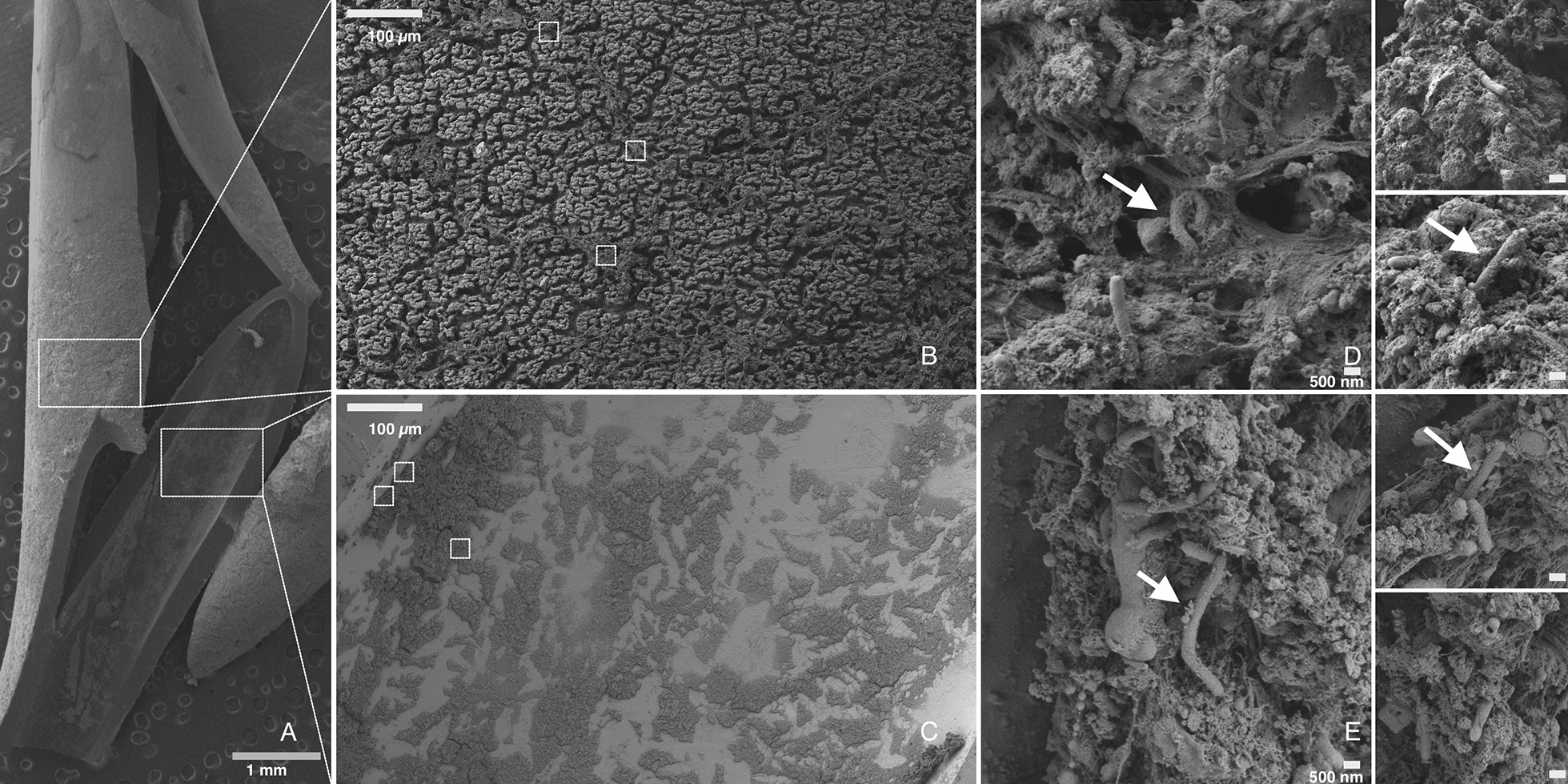
Electron microscopy imaging of biofilm catheter infection. Electron microscopy image of the central venous catheter (**A**) and the biofilm formed on the external (**B**) and internal (**C**) surface. The biofilm is visible as a fibrous complex with rod-shaped bacteria (white arrows) (**D**, **E**). The white bar represents the scaling and is 1 mm in **A**, 100 µm in **B** and **C**, and 500 nm in **D** and **E**, respectively

By means of next-generation sequencing (NGS) of cell-free DNA (Noscendo DISQVER, Duisburg, Germany), *M. chimaera* was detected in blood samples obtained via the port-catheter. Subsequently, specific mycobacterial diagnostics were initiated. *M. chimaera* was recovered from mycobacterial cultures from heparin blood using Mycobacteria growth indicator tube (MGIT) (time to positivity 7 days and 11 h for the first culture and 7 days and 20 h for the second) obtained via the port-catheter but not from peripheral blood and identified by GenoType CM 2.0 and GenoType NTM-DR (Hain Lifescience, Nehren, Germany). *M. chimaera* was also recovered from solid culture media (Löwenstein-Jensen and Stonebrink, Oxoid, Wesel, Germany). After the port-catheter was removed, electron microscopy illustrated the presence of rod-shaped bacteria (consistent with mycobacteria) and a biofilm on the inner and outer surfaces of the port-catheter (Fig. 1). Following diagnosis of NTM bloodstream infection, formalin-bedded hepatic tissue was re-investigated for NTM by PCR, which yielded negative results.

Further immunologic and genetic work-up including inter alia autoimmune markers, immune cell phenotyping and a genetic panel for inherited immunodeficiencies revealed no underlying predisposition to mycobacterial infections apart from CF. Serum levels of pro-inflammatory cytokine IL-6 were normal, sIL-2 receptor levels were mildly elevated (1414 U/ml, cut-off: 700 U/ml), interferon gamma levels were also elevated (298.8 pg/ml, cut-off 30.2 pg/ml).

After removing the port catheter and initiating triple antimycobacterial therapy according to US and European consensus guidelines (rifampicin, ethambutol and azithromycin) [14], the patient’s condition and lung function quickly recovered completely. The liver enzymes were normalized a week after initiation of MAC therapy. The patient received antimycobacterial therapy for 8 weeks, which was then discontinued in favor of CFTR-modulator therapy incompatible with rifamycins due to CYP3A interactions. The sputum cultures remained negative for *M. chimaera* after catheter removal and start of targeted therapy. In the follow-up over months after the end of the therapy there were no signs of infection relapse but CFTR-modulator therapy was dose-adjusted due to relapse of elevated liver enzymes.

## Discussion

The ability of MAC to cause biofilm infections on plastic material as central vein catheters has been described and investigated in vitro and in vivo. *Falkinham J.* described in-vitro the ability of MAC to cause biofilm on plastic material, such as vein catheters [15]. In this study, biofilm infections were associated with high resistance to the common antibiotics used against MAC (clarithromycin, rifamycin), whereas other in-vitro studies showed lower susceptibility for rifampin and ethambutol contrary to other antibiotics (such as clarithromycin, amikacin, rifabutin, and streptomycin) [16]. However, in-vivo, only a few cases of MAC catheter-associated biofilm infections have been described, and all of those were in the context of immunosuppression [17]. In immunocompetent patients, MAC is considered a low-virulent pathogen that causes localized infections of the lung or soft tissue.

The largest retrospective study on epidemiology and diagnosis of mycobacterial infections in children included 105 patients. In this cohort only 11 bloodstream infections were identified of whom 7 were catheter-associated with all affected patients receiving immunosuppressive therapy. Notably, all of the infections were caused by rapidly-growing NTM [18]. In another retrospective study, *Al Yazidi *et al. described a series of 54 pediatric patients with NTM disease, among those 8 patients had bacteremia caused by rapidly-growing NTM all associated with a central venous catheter, again all in association with impaired immunocompetence. In this study, sole catheter removal without or with a short subsequent NTM-targeted pharmacotherapy led to cure of all patients [19]. The published data reveal an association between NTM bacteremia and immunodeficiency and emphasize the importance of the catheter removal in case of catheter-associated infection.

We present the first case of catheter-associated MAC infection in an immunocompetent and/or CF individual. This case highlights the importance for active search for NTM infections as conventional blood cultures for bacteria and fungi are typically non-diagnostic. In the context of an unremarkable chest CT scan and thus unmet criteria of NTM pulmonary disease [11], we did not attribute the systemic symptoms or granulomatous hepatitis to the *Mycobacterium chimaera* recovered from sputum samples. Therefore, targeted therapy of MAC pulmonary disease, which can also be hepatotoxic, was not initiated. Notably, the etiology of the hepatitis was not completely clarified in our patient. We initially did not draw heparin blood for mycobacteria cultures, until NGS guided to the rare and unexpected diagnosis. NGS has the enormous advantage that no specific primers are required, so that virtually every DNA genome information can be detected within a single analysis. NGS has been shown to help in the identification of rare microorganisms such as NTM and was a crucial diagnostic step in this case [20].

## Conclusion

MAC needs to be considered as a cause of bloodstream infections originating from biofilms on long-term venous catheters even in immunocompetent patients. Next generation sequencing represents a powerful and quick diagnostic tool. The crucial therapeutic step is the removal of the catheter.

## Data Availability

Not applicable.
